# Circular RNA circ-ITCH inhibits bladder cancer progression by sponging miR-17/miR-224 and regulating p21, PTEN expression

**DOI:** 10.1186/s12943-018-0771-7

**Published:** 2018-01-31

**Authors:** Chengdi Yang, Wenbo Yuan, Xiao Yang, Peng Li, Jingzi Wang, Jie Han, Jun Tao, Pengchao Li, Haiwei Yang, Qiang Lv, Wei Zhang

**Affiliations:** Department of Urology, The First Affiliated Hospital of Nanjing Medical University, Nanjing, 210029 People’s Republic of China

**Keywords:** circ-ITCH, Bladder cancer, miR-17, miR-224, p21, PTEN

## Abstract

**Background:**

Circ-ITCH is a circRNA generated from several exons of itchy E3 ubiquitin protein ligase (ITCH) and tumor suppressor served as a sponge for certain miRNAs targeting their parental transcripts of ITCH. However, the role of circ-ITCH in bladder cancer (BCa) was not reported. In the present study, we investigated the role of circ-ITCH in BCa.

**Methods:**

Quantitative real-time PCR was used to detect the expression of circ-ITCH and survival analysis was adopted to explore the association between circ-ITCH expression and the prognosis of BCa. BCa cells were stably transfected with lentivirus approach and cell proliferation, migration, invasion, cell cycle and cell apoptosis, as well as tumorigenesis in nude mice were performed to assess the effect of circ-ITCH in BCa. Biotin-coupled probe pull down assay, Biotin-coupled miRNA capture, Fluorescence in situ hybridization and Luciferase reporter assay were conducted to confirm the relationship between the circ-ITCH and the microRNA.

**Results:**

In the present study, we found that circ-ITCH, is down-regulated in BCa tissues and cell lines. BCa patients with low circ-ITCH expression had shortened survival. Enforced- expression of circ-ITCH inhibited cells proliferation, migration, invasion and metastasis both in vitro and in vivo. Mechanistically, we demonstrated that circ-ITCH up-regulates the expression of miR-17 and miR-224 target gene p21 and PTEN through ‘sponging’ miR-17 and miR-224, which suppressed the aggressive biological behaviors of BCa.

**Conclusions:**

circ-ITCH acts as a tumor suppressor by a novel circ-ITCH/miR-17, miR-224/p21, PTEN axis, which may provide a potential biomarker and therapeutic target for the management of BCa.

**Electronic supplementary material:**

The online version of this article (10.1186/s12943-018-0771-7) contains supplementary material, which is available to authorized users.

## Background

Bladder cancer (BCa) has become one of the most prevalent malignancies worldwide, with an estimated 430,000 new cases diagnosed in 2012 [1]. Surgery, chemotherapy and radiation therapy are the mainstay treatments for BCa patients. However, the recurrence and progression rates are still high and the 5-year cancer-specific survival rate remains at a low level [2, 3]. One important reason is the poor understanding of the mechanisms underlying BCa development and progression. Therefore, detailed researches into the molecular mechanisms are essential for improving the diagnosis, prevention and treatment of this disease.

Circular RNA (circRNA) is a novel class of endogenous no-coding RNAs formed by a covalently closed loop [4, 5]. Previous studies revealed that circRNAs were differentially expressed in various cancerous tissues or cell lines, and tightly correlated with the disease status or prognosis [6]. Concretely, circRNAs are involved in the occurrence of many cancers such as hepatocellular carcinoma (HCC), gastric cancer, colorectal cancer and so on, by serving as microRNA (miRNA) sponges and protecting the target genes from miRNA-mediated mRNA cleavage. These circRNA-miRNA regulatory networks work on target genes involved in cell cycle regulation, signal transduction, epigenetic modulation or transcriptional regulation, which ultimately regulate cancer cell proliferation, differentiation, invasion and metastasis [7]. Moreover, some circRNAs have the potential to be valuable candidates as biomarkers or prognosis factors in tumors [8]. However, there are currently few reports describing the role of circRNAs in BCa except the circTCF25 [9]. The biological or pathological functions of individual circRNA in BCa remain largely unknown and need further investigation.

Circ-ITCH is a well-known circRNA generated from several exons of itchy E3 ubiquitin protein ligase (ITCH), which was firstly covered in the study by Memczak et al. [4, 10]. Circ-ITCH was reported to be downregulated in colorectal cancer [11], esophageal squamous cell carcinoma [12], lung cancer [13] and hepatocellular carcinoma [14], and a powerful tumor suppressor among through a classic pathway in which circ-ITCH served as a sponge for certain miRNAs targeting their parental transcripts of ITCH [11–13]. Therefore, circ-ITCH protected ITCH and blocked the downstream Wnt/β-catenin signaling, which resulted in arresting tumor progression [11–13]. However, the role of circ-ITCH in BCa was not reported. More important, whether there exists other regulatory axis for circ-ITCH regulating tumor progression, besides the classic pathway above, was still unknown. As a cyclin-dependent kinase inhibitor or famous tumor suppressor individually, p21 and phosphatase and tensin homologue (PTEN) was validated to be key molecules in regulating BCa. The loss of p21 was associated with BCa progression, poor prognosis, high recurrence and frustrated survival while PTEN deletion was associated with aggressive tumor phenotype and adverse prognosis, both of which are expected to be crucial therapeutic target for BCa [15–17]. Additionally, early studies demonstrated that multiple oncogenic miRNAs promoted malignancy in tumors by neutralizing p21 or PTEN expression [18–21]. Considering that circRNA exerts its function mainly by acting as miRNA sponge currently, we speculated that p21 and PTEN could be mediately regulated via the sponge function of circRNA in BCa. Coincidently, by bioinformatic analysis (Starbase V2.0 [22], Circinteractome [23]), we found that both circ-ITCH and the 3′-untranslated region (UTR) of p21, PTEN share miRNA response elements (MREs) of miR-17 and mir-224, which suggested the association between circ-ITCH and p21/ PTEN in BCa.

Therefore, we presented a hypothesis that circ-ITCH might be involved in BCa progression by influencing the expression of p21 and PTEN in a miRNA-mediated manner. Biological and molecular experiments in vitro or in vivo were conducted to address this speculation. Moreover, we analyzed the expression of circ-ITCH in BCa samples to explore its diagnosis and prognostic potential in BCa.

## Methods

### Tissue samples

BCa tumor and normal tissues were obtained from patients who were diagnosed with BCa and undergone surgery in the first affiliated hospital of Nanjing Medical University between 2013 and 2016. In total, 72 pairs of tissue samples were freshly frozen in liquid nitrogen and stored at − 80 °C until RNA extraction. The use of tissues for this study has been approved by the ethics committee of the first affiliated hospital of Nanjing Medical University. All patients signed the informed consent before using the clinical materials for research purposes. Additionally, a cohort of 70 BCa patients with clinicopathological parameters was followed up. The follow-up time ranged from 1 to 60 months. The follow-up interval began on the date of surgery and ended on the date of disease progression.

### Cell culture and transfection

The BCa cell lines were purchased from the Type Culture Collection of the Chinese Academy of Sciences (Shanghai, China) and cultured with DMEM medium (Gibco, USA) containing 10% fetal bovine serum (FBS). The cells were cultured at 37 °C in a humidified incubator containing 5% CO_2_. To overexpress circ-ITCH, a fragment of 873 bp of cDNA was cloned into PLCDH-cir vector (Ribobio, Guangzhou, China) and lentivirus was constructed by Hanbio (Shanghai, China). The procedure of transfection was conducted as previously described according to the manufacturer’s instructions [24].

### RNA extraction and quantitative real-time polymerase chain reaction (qRT-PCR)

Total RNA was isolated from tissues and cells by using Trizol reagent (Invitrogen, USA) according to the manufacturer’s protocol. cDNA was synthesized using HiScript II (Vazyme, China) and qRT-PCR for circRNA and miRNA was performed on AB7300 thermo-recycler (Applied Biosystems, USA) or LightCycler 480 (Roche, USA) using primers (Sango Biotech, China) listed in Additional file 1 Table S1. U6 and β-actin were used as an internal standard control for miRNA and mRNA detection, respectively. Each sample was replicated three times and data was analyzed by comparing Ct values.

### Cell proliferation assay and cloning formation assay

For cell proliferation assay, the transfected cells were seeded into 96-well plates at a density of 2000 cells per well. At 0, 24, 48, 72 and 96 h after seeding, cell viability was measured by the cell counting kit-8 (CCK-8) system (Dojindo, Japan) according to the manufacturer’s instructions. Briefly, each well was added with 10 μl CCK-8 solution and the plate was incubated at 37 °C for 1 h in dark. Absorbance at 450 nm of each well was measured using a microplate reader (Tecan, Switzerland).

For colony formation assay, the transfected cells were seeded into 6-well plates at a density of 400 cells per well and maintained in DMEM medium containing 10% FBS. After two weeks, the cells were fixed with methanol and staining with 0.1% crystal violet and the colonies were imaged and counted.

### Migration and invasion assays

In this assay, about 1 × 10^4^ transfected cells were suspended in 200 μl of serum-free medium and seeded into the upper chambers of each transwell (8 μm pore size, Costar), which was coated with or without Matrigel (BD Biosciences, USA) for the invasion and migration assays. Medium containing 10% FBS was added to the bottom chamber as a chemoattractant. The cells were incubated at 37 °C with 5% CO_2_ for 48 h for the invasion assay and 24 h for the migration assay. After incubation, cells in the top chamber were removed with cotton swabs and the cells on the lower surface were fixed with methanol, stained with 0.1% crystal violet, and photographed under a microscope at × 100 magnification (Olympus, Japan).

### Cell cycle and apoptosis assays

For the cell cycle analysis, the transfected cells were stained with propidium iodide by the cycletest plus DNA reagent kit (BD Biosciences, USA) and then measured by flow cytometry (Attune NxT, Thermo Fisher, USA). The ratios of cells in the G0/G1, S, G2 phases were counted and compared. To detect cell apoptosis, cells were stained using an annexin V-APC/7AAD apoptosis kit (eBiosciences, USA) and analyzed using flow cytometry. The ratio of early apoptotic cells to late apoptotic cells was compared to the values obtained for the controls in each experiment.

### Western blot

Cells were lysed with radio immunoprecipitation assay buffer (RIPA, Beyotime, China), and protein was harvested and quantified by bicinchoninic acid (BCA) analysis (Beyotime, China). Protein extractions were separated by 10% SDS-PAGE and transferred onto polyvinylidene fluoride (PVDF) membranes (Sigma-Aldrich, USA). After the incubation with a high affinity anti-p21 antibody (1:1000), anti-PTEN antibody (1:1000), anti-β-actin antibody (1:2000) (Cell Signaling Technology, USA), the membranes were then incubated with a secondary antibody (1:5000, Cell Signaling Technology, USA). After washes, signals were detected using a chemiluminescence system (Bio-Rad, USA) and analyzed using Image Lab Software.

### Biotin-coupled probe pull down assay

The biotinylated probe was specifically designed to bind to the junction area of circ-ITCH, while oligo probe was taken as control. About 1 × 10^7^ cells were washed in ice-cold phosphate-buffered saline (PBS), lysed in lysis buffer and incubated with 3 μg biotinylated probes at room temperature for 2 h. The biotin-coupled RNA complex was pull-downed by incubating the cell lysates with streptavidin magnetic beads (Life Technologies, USA) for another 4 h. The beads were washed with lysis buffer for five times and the bound miRNAs in the pull-down materials were extracted using Trizol reagent and analyzed by qRT-PCR assay.

### Biotin-coupled miRNA capture

Approximately 2 × 10^6^ cells were transfected with 50 μM of biotinylated miRNA mimics or nonsense control (NC) (GenePharma, Shanghai, China) at 50% confluence for lysis. At 24 h after transfection, the cells were harvested and washed in PBS, lysed in lysis buffer. A total of 50 μl washed streptavidin magnetic beads were blocked for 2 h and then added to each reaction tube to pull down the biotin-coupled RNA complex. All the tubes were incubated for 4 h on the rotator at a low speed (10r /min). The beads were washed with lysis buffer for five times and Trizol LS (Life Technology, USA) was used to recover RNAs specifically interacting with miRNA. The abundance of circ-ITCH in bound fractions was evaluated by qRT-PCR analysis and agarose gel electrophoresis.

### Northern blot

Northern blot analysis was performed with northern blot kit (Ambion, USA). Briefly, about total RNAs (30 μg) was denatured in formaldehyde and then electrophoresed in a 1% agarose–formaldehyde gel. The RNAs were then transferred onto a Hybond-N + nylonmembrane (Beyotime, China) and hybridized with biotin-labeled DNA probes. Biotin chromogenic detection kit (Thermo Scientific, USA) was used to detect the bound RNAs. Finally, the membranes were exposed and analyzed using Image Lab software (Bio Rad, USA).

### Fluorescence in situ hybridization (FISH)

Specific probes to circ-ITCH sequence and miR-17/miR-224 was used in situ hybridization. In brief, cy5-labelled probes were specific to circ-ITCH and farm-labelled probes were specific to miRNAs. Nuclei were counter by staining with 4,6-diamidino-2-phenylindole (DAPI). All the procedures were conducted according to the manufactory’s instruction (Genepharma, Shanghai, China). All images were acquired on Zeiss LSM880 NLO (2 + 1 with BIG) confocal microscope system (Leica Microsystems, Mannheim, Germany).

### Luciferase reporter assay

Cells were co-transfected with plasmids containing 3′-UTR of wild or mutant fragments from p21/ PTEN and miRNA mimics using Lipofectamine3000 (Invitrogen, Foster city, CA) according to the manufacturer’s protocol. 24 h after transfection, firefly and renilla luciferase activities were measured consecutively by using dual-luciferase reporter assay system (Promega, Massachusetts, USA). Finally, ratios of luminescence from firefly to renilla luciferase were calculated and each assay was repeated in 3 independent experiments.

### Xenografts in mice

The T24 cells were stably transfected with circ-ITCH and GFP vector. About 1× 10^7^ cells were injected subcutaneously into the axilla of the female athymic BALB/C nude mice (4–6 weeks old, 18–22 g, five mice per group). Tumor growth was monitored every week by measuring the width (W) and length (L) with calipers, and the volume (V) of the tumor was calculated using the formula V = (W2 × L)/2. Four weeks after injection, the mice were euthanized and tumor weights were measured. The animal studies were performed in accordance with the institutional ethics guidelines for animal experiments, which was approved by the animal management committee of Nanjing Medical University.

### Immunohistochemistry (IHC) analysis

The paraffin-embedded tissues from nude mice were cut into 4 μm slides. Rabbit p21 and PTEN antibody were purchased from Cell Signaling Technology Company. IHC analysis was performed according to the procedure described previously [23]. The images were obtained under a microscope (Olympus, Japan) with appropriate magnification.

### Statistical analysis

All the analyses were performed with SPSS 19.0 (IBM, SPSS, Chicago, IL, USA) and *p*-value < 0.05 was considered to be statistically significant. Data differences between two groups were analyzed using the Student’s t test or Chi-square test. The Pearson’s correlation coefficient analysis was used to analyze the correlations. Survival analysis was performed by Kaplan-Meier curves and log-rank test for significance.

## Results

### Circ-ITCH was decreased in BCa and correlated with prognosis of BCa patients

**Fig. 1:**
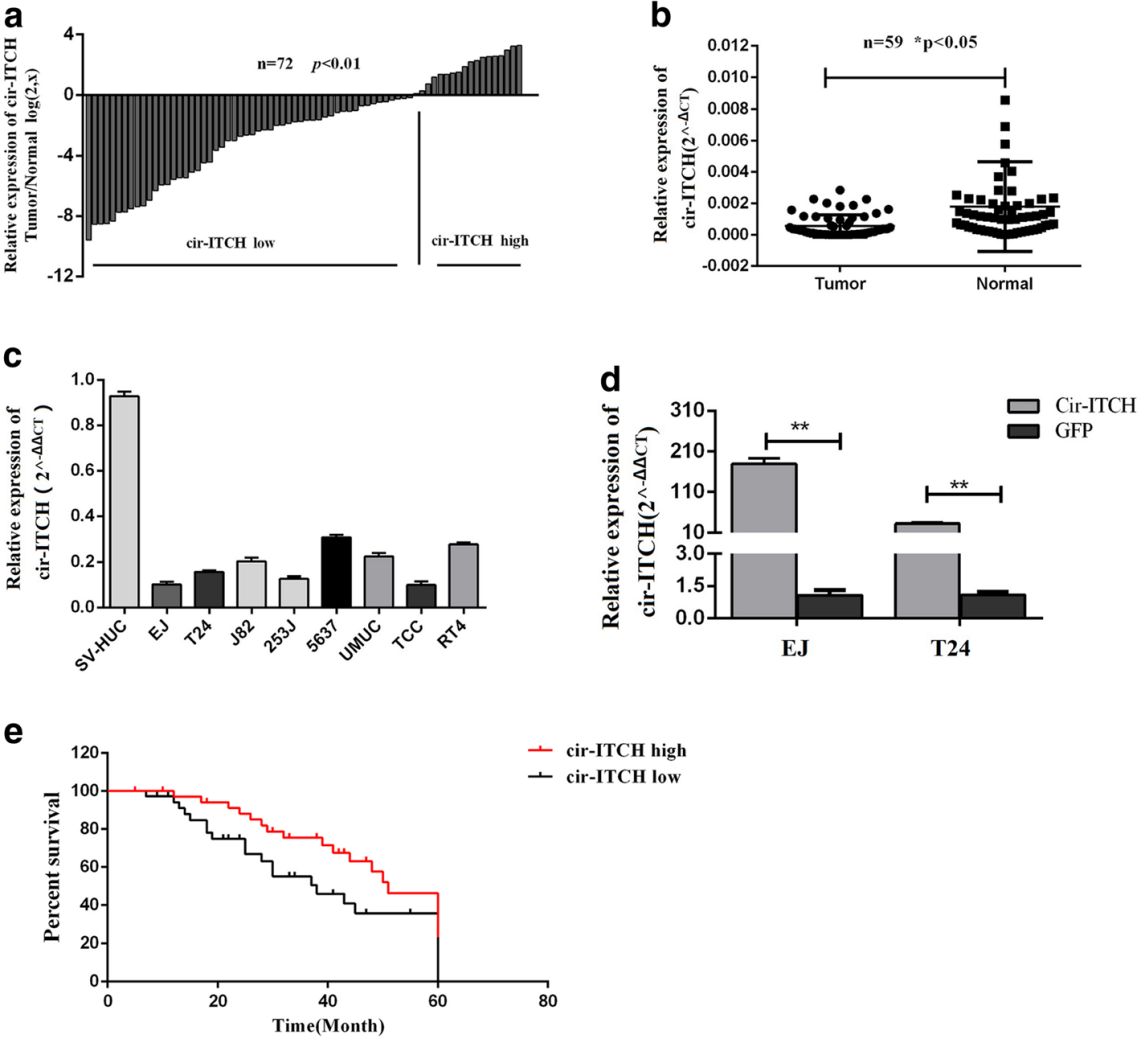
Cir-ITCH was significantly decreased in BCa and correlated with prognosis of BCa patients. **a** and **b** Relative expression level of cir-ITCH in BCa tissues (T) and adjacent normal tissues (N) (a: *n* = 72, b: *n* = 59) using qRT-PCR. cir-ITCH expression was significantly lower in bladder cancer tissues, compared with that in adjacent normal tissues (*P < 0.05, Student’s *t*-test). **c** Relative expression level of in bladder cancer cell lines and SV-HUC cell using qRT-PCR. **d** qRT-PCR confirmed the over-expression of cir-ITCH in BCa cell lines EJ and T24 after transfection (*P < 0.05, **P < 0.01, Student’s *t*-test). **e** Kaplan-Meier plotter analysis of the correlation of circ-ITCH expression level with overall survival of BCa patients

To investigate whether the expression of circ-ITCH was altered in BCa, we firstly examined the expression of circ-ITCH in 72 pairs of BCa tissues and matched adjacent normal tissues using qRT-PCR. The result revealed that the expression of circ-ITCH was significantly decreased in BCa tissues, compared to that in the matched adjacent normal tissues (Fig. 1a, b). Also, circ-ITCH showed a lower expression in 8 BCa cell lines (EJ, T24, 253 J, RT4, TCC-SUP, UMUC, J82, 5637), compared to SV-HUC, which is a normal urothelial cell line (Fig. 1c).

Additionally, a cohort of 70 BCa patients with clinicopathological parameters and survival data was included to analyze whether circ-ITCH is correlated with the prognosis. We found the level of circ-ITCH expression was positively correlated with histological grade (*P* = 0.034), but not with other clinicopathological characteristics, including age, tumor node metastasis stage (TNM) and tumor size (Table 1). Additionally, BCa patients with lower circ-ITCH expression had a worse overall survival than patients with higher circ-ITCH expression (Fig. 1e). Together, we found that lower circ-ITCH expression led to a worse prognosis in BCa patients.

**Table 1:** Correlations between the proportion of cir-ITCH and clinicopathological features in 70 BCa patients

Characteristics	Case	cir-ITCH expression		*P* value
		Low	High	
All cases	70	45	25	
Age(years)				0.443
< 65	26	15	11	
≥ 65	44	30	14	
Gender				0.765
Male	55	36	19	
Female	15	9	6	
TNM stage				0.186
pTa-pT1	23	12	11	
pT2-pT4	47	33	14	
Histological grade				0.034*
Low	24	11	13	
High	46	34	12	
Tumor size(cm)				0.140
< 3	36	20	16	
≥ 3	34	25	9	

*P < 0.05

### Circ-ITCH inhibited the progression of BCa cells in vitro

**Fig. 2:**
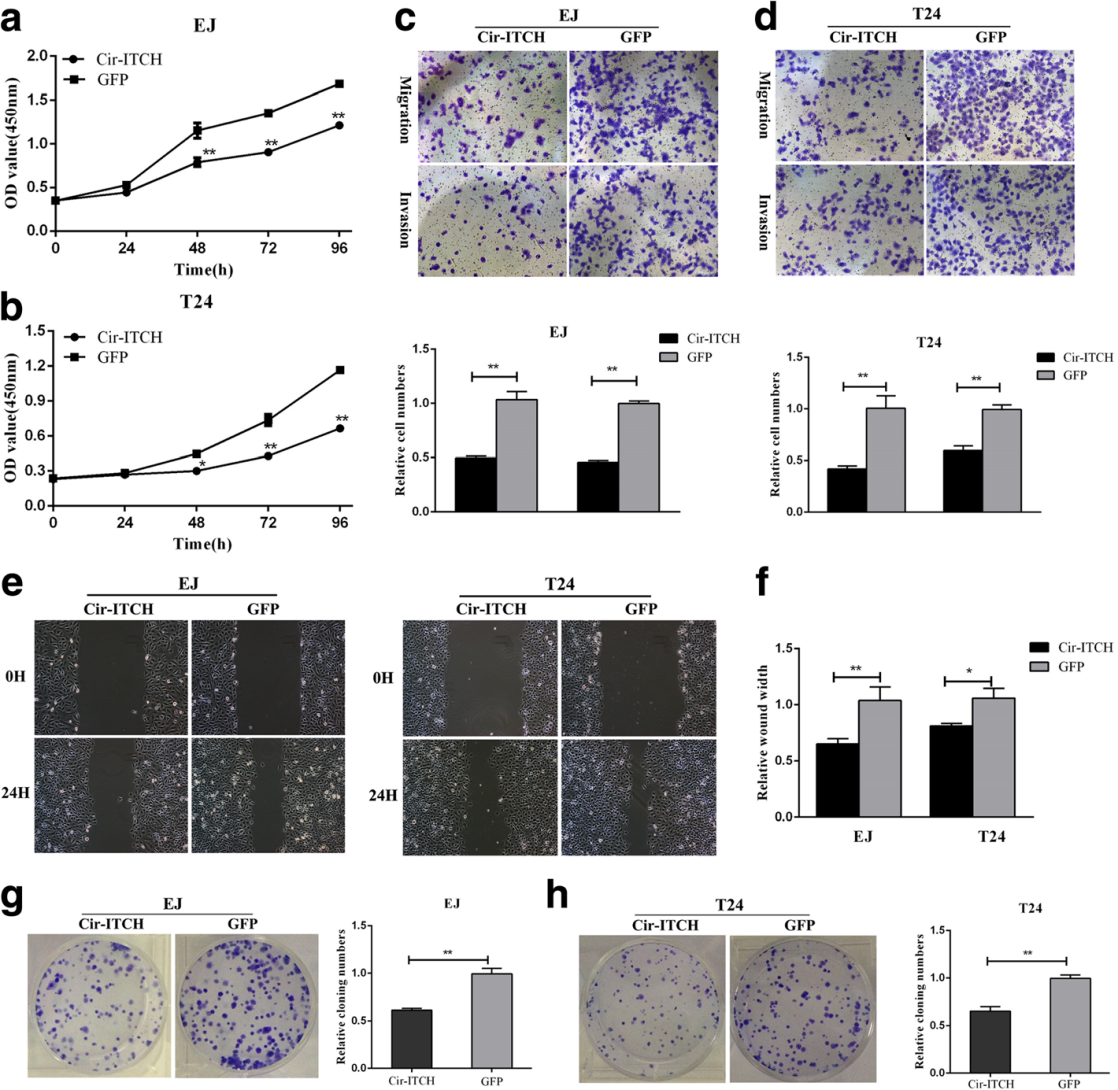
Over-expression of cir-ITCH inhibits bladder cancer cell growth, colony formation, migration and invasion in vitro. **a** and **b** Cir-ITCH inhibited cell proliferation as indicated by CCk-8 assays in EJ and T24 cells. Data are mean ± S.D. from triplicate experiments (*P < 0.05, **P < 0.01, Student’s *t*-test). **c** and **d** Transwell invasion assay was measured and the results were expressed as the number of invaded cells per field compared with respective control (magnification, × 100, *P < 0.05, Student’s *t*-test). **e** and **f** Wound healing assay showed that cir-ITCH resulted in a slower closing of scratch wounds (*P < 0.05, **P < 0.01, Student’s *t*-test). **g** and **h** Cir-ITCH inhibited the colony formation of EJ and T24 cells as demonstrated by colony formation assays (*P < 0.05, **P < 0.01, Student’s *t*-test)

Given that circ-ITCH is down-regulated in BCa tissues and cell lines in our study, we further investigated its potential functional role by overexpressing circ-ITCH in EJ and T24 cell lines. As shown in Fig. 1d and Additional file 2 Figure S1a, qRT-PCR and northern blot confirmed the overexpression efficiency of circ-ITCH, which had no obvious effect on the expression of its parental gene ITCH (Additional file 2 Figure S1b). Functionally, CCK-8 assays revealed that the viability of EJ and T24 was decreased in circ-ITCH overexpressing group compared with that in GFP group (*P* < 0.01). Consequently, colony numbers of circ-ITCH overexpressing cells were significantly less than those of GFP group (*P* < 0.05, Fig. 2g, h). Meanwhile, transwell migration and invasion assays indicated that the migration and invasion abilities of EJ and T24 cell lines were also suppressed by circ-ITCH, which was in accordance to the wound healing assays (*P* < 0.01, Fig. 2c, d).

**Fig. 3:**
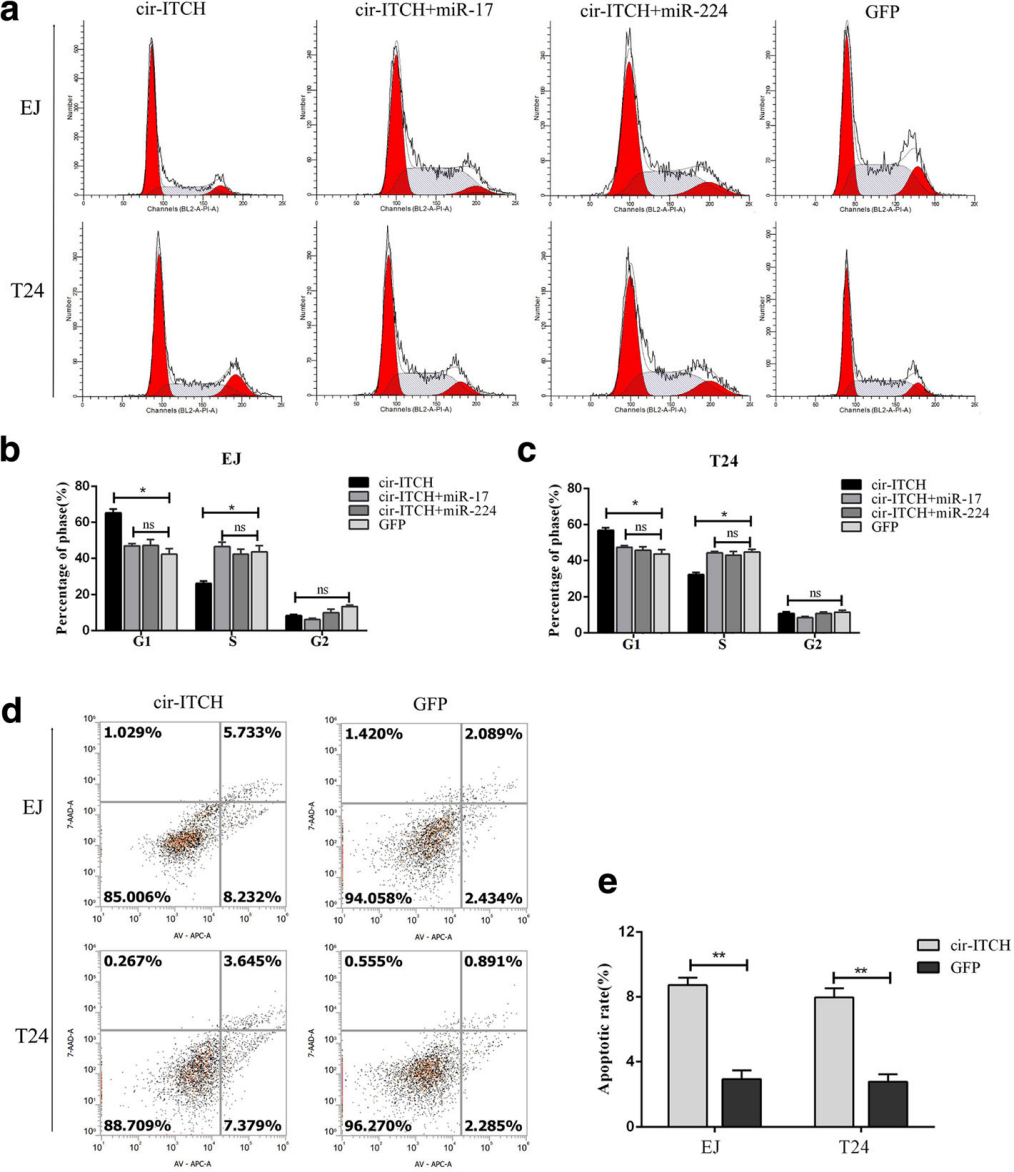
Cir-ITCH induced cell cycle arrest and cell apoptosis in BCa cells. **a** Representative images of the cell cycle analysis using flow cytometry. **b** Cir-ITCH arrested the EJ and T24 cell cycle at the G1/S phase, while miR-17 and miR-224 turned the G1/S transition. **c** Representative images of Annexin V and 7-AAD staining apoptosis assay in EJ and T24 cells. **d** and **e** Apoptosis assay showed that increased the rate of apoptosis in EJ and T24 cells. Data are the means ± SEM of three experiments, (*P < 0.05, **P < 0.01, Student’s *t*-test)

Flow cytometry analysis was performed to evaluate whether circ-ITCH affect BCa cells phenotype by altering the cell cycle profile and apoptosis. As shown in Fig. 3a, more cells were distributed in G1 phase after overexpression of circ-ITCH, which suggested that circ-ITCH induced G1/S cell cycle arrest. In addition, apoptosis assays also revealed that circ-ITCH has an apoptosis-inducing character in BCa cells (*P* < 0.05, Fig. 3d, e). These data suggested that Circ-ITCH inhibited the progression of BCa cells in vitro.

### Circ-ITCH acts as a molecular sponge for miR-17 and miR-224

**Fig. 4:**
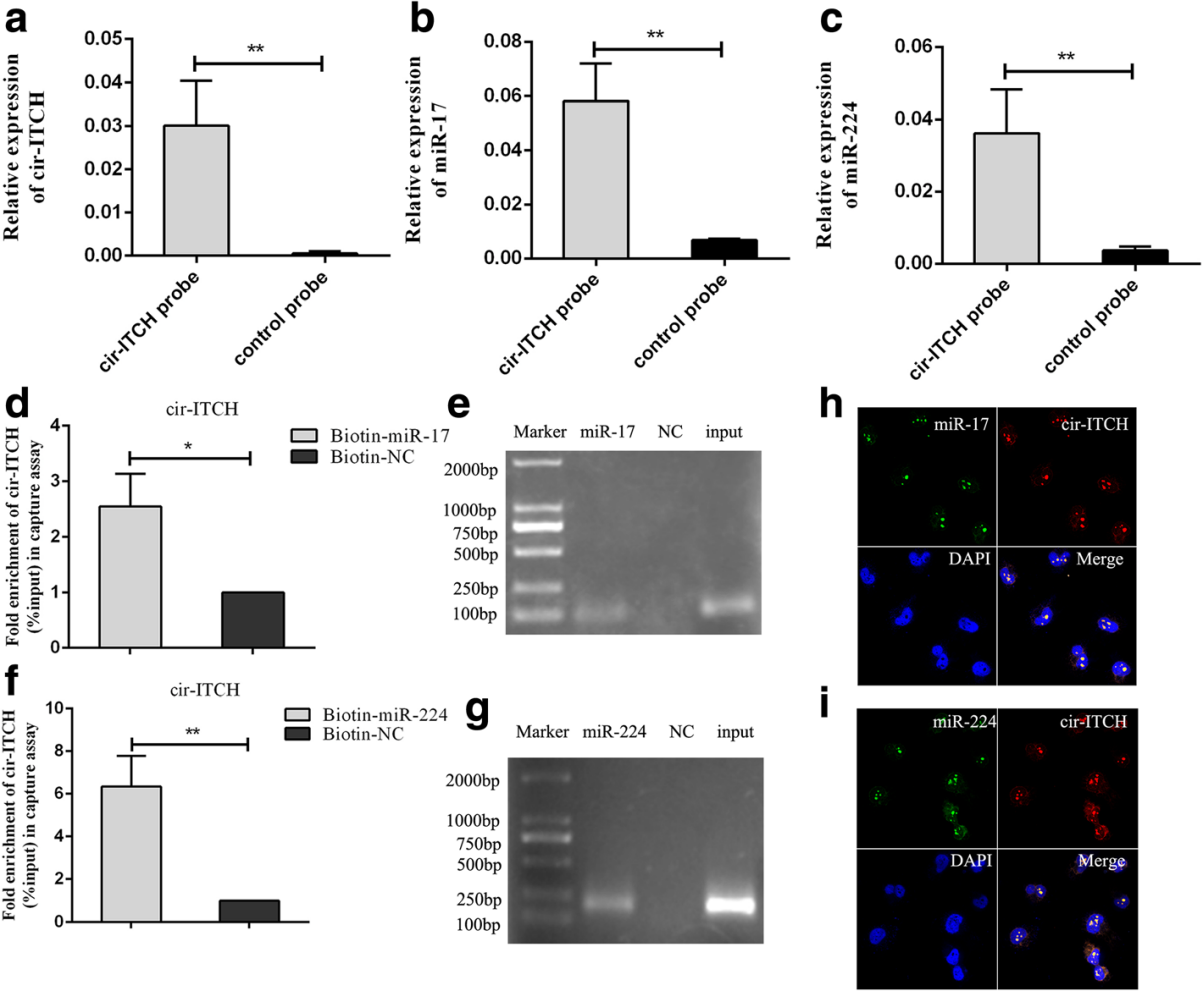
Cir-ITCH acted as a sponge for miR-17 and miR-224. **a** Cir-ITCH in BCa cell lysis was pulled down and enriched with cir-ITCH specific probe and then detected by qRT-PCR(**P < 0.01, Student’s *t*-test). **b** miR-17 was pulled down and enriched with cir-ITCH specific probe and then detected by qRT-PCR(**P < 0.01, Student’s *t*-test). **c** miR-224 was pulled down and enriched with cir-ITCH specific probe and then detected by qRT-PCR(**P < 0.01, Student’s *t*-test). **d** and **f** Biotin-coupled miR-17/miR-224 captured a fold change of cir-ITCH in the complex as compared with biotin-coupled NC in biotin-coupled miRNA capture(*P < 0.05, **P < 0.01, Student’s *t*-test). **e** and **g** The product of d and f was detected using qRT-PCR, followed by agarose gel electrophoresis. **h** and **i** RNA FISH for cir-ITCH and miR-17/miR-224 was detected in BCa cells, miR-17 and miR-224 were co-localized with cir-ITCH in cytoplasm (magnification, × 400). Nuclei was stained blue (DAPI), cir-ITCH was stained red, miR-17/miR-224 was stained green

CircRNAs function mainly as miRNA sponges to bind functional miRNAs and then regulate gene expression [24]. In this study, by bioinformatic analysis (Starbase V2.0, Circinteractome), we found that circ-ITCH shares miRNA response elements (MREs) of miR-17 and mir-224, which might possibly bind with circ-ITCH in BCa. Subsequently, we applied the biotin-coupled probe pull down assay to further confirm this interaction. As shown in Fig. 4b, c, a specific enrichment of circ-ITCH, miR-17 and miR-224 was detected in the circ-ITCH pulled down pellet compared with control group, demonstrating that circ-ITCH could directly sponge miR-17 and miR-224. To further confirm the sponge effect of circ-ITCH, we conducted the biotin-coupled miRNA capture and FISH. Analogously, biotin-coupled miR-17/miR-224 captured more circ-ITCH than biotin-coupled NC, indicating that miR-17/miR-224 could bind to circ-ITCH (Fig. 4d, f). Meanwhile, FISH analysis in BCa cells showed that circ-ITCH was co-localized with miR-17 or miR-224 in the cytoplasm (Fig. 4h, i).

**Fig. 5:**
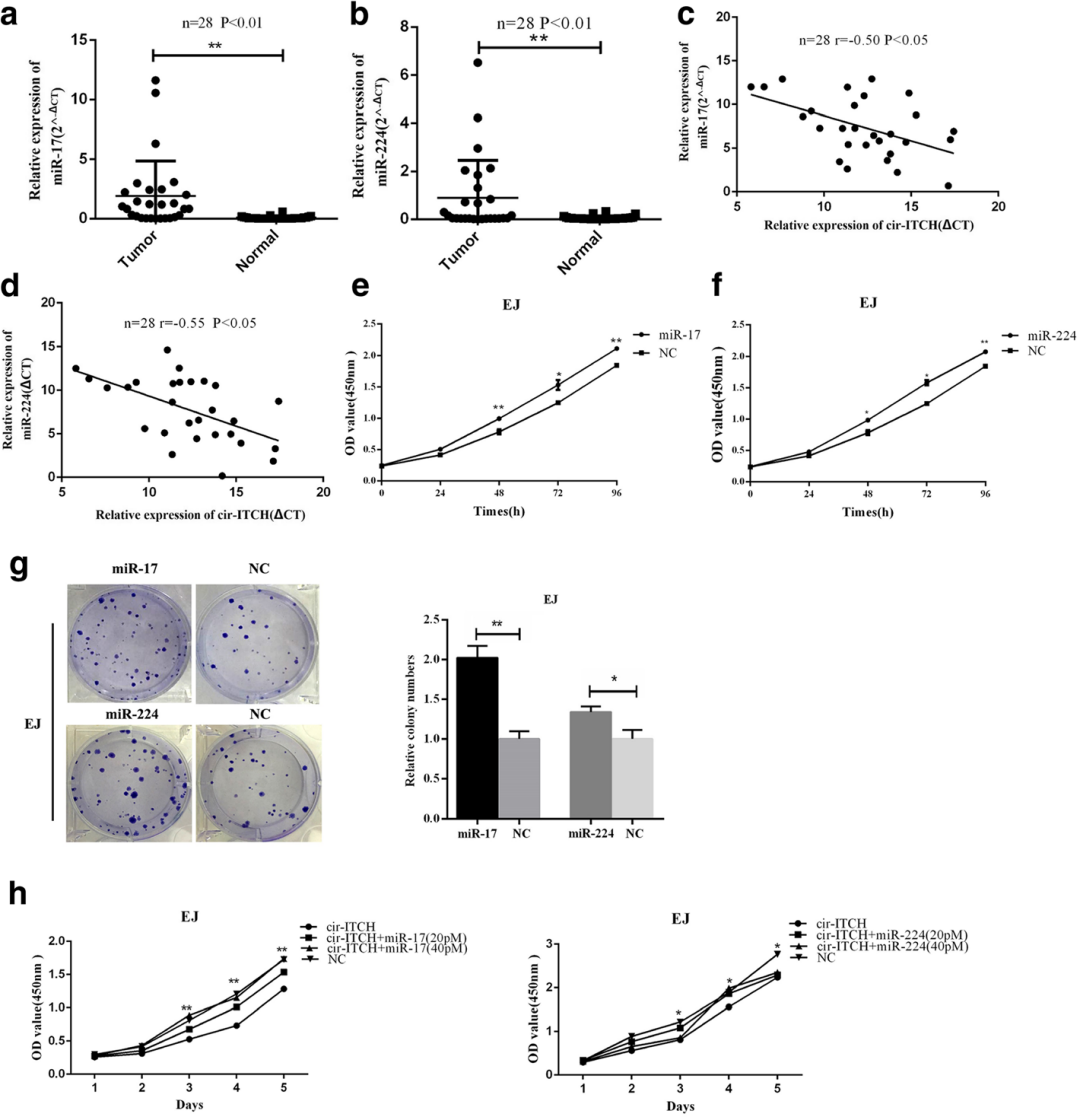
miR-17 and miR-224 played as oncogenes in BCa and eliminated the repression function of cir-ITCH. **a** and **b** miR-17 and miR-224 were up-regulated in BCa tissues as compared with adjacent normal tissues using qRT-PCR (*n* = 28, **P < 0.01, Student’s *t*-test). **c** and **d** A moderate negative correlation between the expression of cir-ITCH and miR-17/miR-224 was showed using Pearson correlation analysis. **e** and **f** CCK-8 assay showed that miR-17 and miR-224 promoted the vitality of EJ cell (*P < 0.05, **P < 0.01, Student’s *t*-test). **g** Cloning formation assay showed that miR-17 and miR-224 increased in cloning number of EJ cell (*P < 0.05, **P < 0.01, Student’s *t*-test). **h** miR-17 and miR-224 reversed the inhibitory effect of cir-ITCH on cell proliferation by CCK-8 assay (*P < 0.05, **P < 0.01, compared to circ-ITCH, Student’s *t*-test.)

Additionally, the expression of miR-17 and miR-224 in 28 pairs of BCa tissues and adjacent normal tissues was measured and both miRNAs were increased in BCa tissues (*P* < 0.05, Fig. 5a, b). Correlation analysis revealed a moderate negative correlation between the expression of circ-ITCH and miR-17(*r* = − 0.50, *P* < 0.01) or miR-224 (*r* = − 0.55, *P* < 0.01) (Fig. 5c, d). Taken together, these results suggest that circ-ITCH acts as a sponge for miR-17 and miR-224 in BCa.

### miR-17 and miR-224 promoted BCa cell proliferation

On the basis of the interaction of circ-ITCH and miR-17/miR-224, we next assessed the potential functional role of miR-17 and miR-224 in BCa by transfecting miRNA mimics. As shown in Fig. 5e, f, enforced expression of miR-17 or miR-224 significantly promoted the viability of cells (*P* < 0.05). Besides, the cloning ability of cells deal with miR-17 or miR-224 mimics were significantly promoted as compared with that in NC group (*P* < 0.05, Fig. 5g). Moreover, rescue experiments were conducted by co-transfecting circ-ITCH and miR-17 (or miR-224) mimics in BCa cells to assess whether the tumor-suppressing effect of circ-ITCH could be blocked by miR-17 (or miR-224) overexpression. As shown in Fig. 5h, miR-17 or miR-224 mimics could partly attenuate the circ-ITCH overexpressing-mediated inhibition of proliferation in BCa cells (*P* < 0.05). Interestingly, with the increasing concentration of miRNA mimics, this effect tended to be more obvious. Above all, these data reflected that circ-ITCH suppressed BCa progression partly via abolishing the oncogenic effect of miR-17/miR-224.

### circ-ITCH modulated the miR-17 (or miR-224) targets p21 and PTEN

**Fig. 6:**
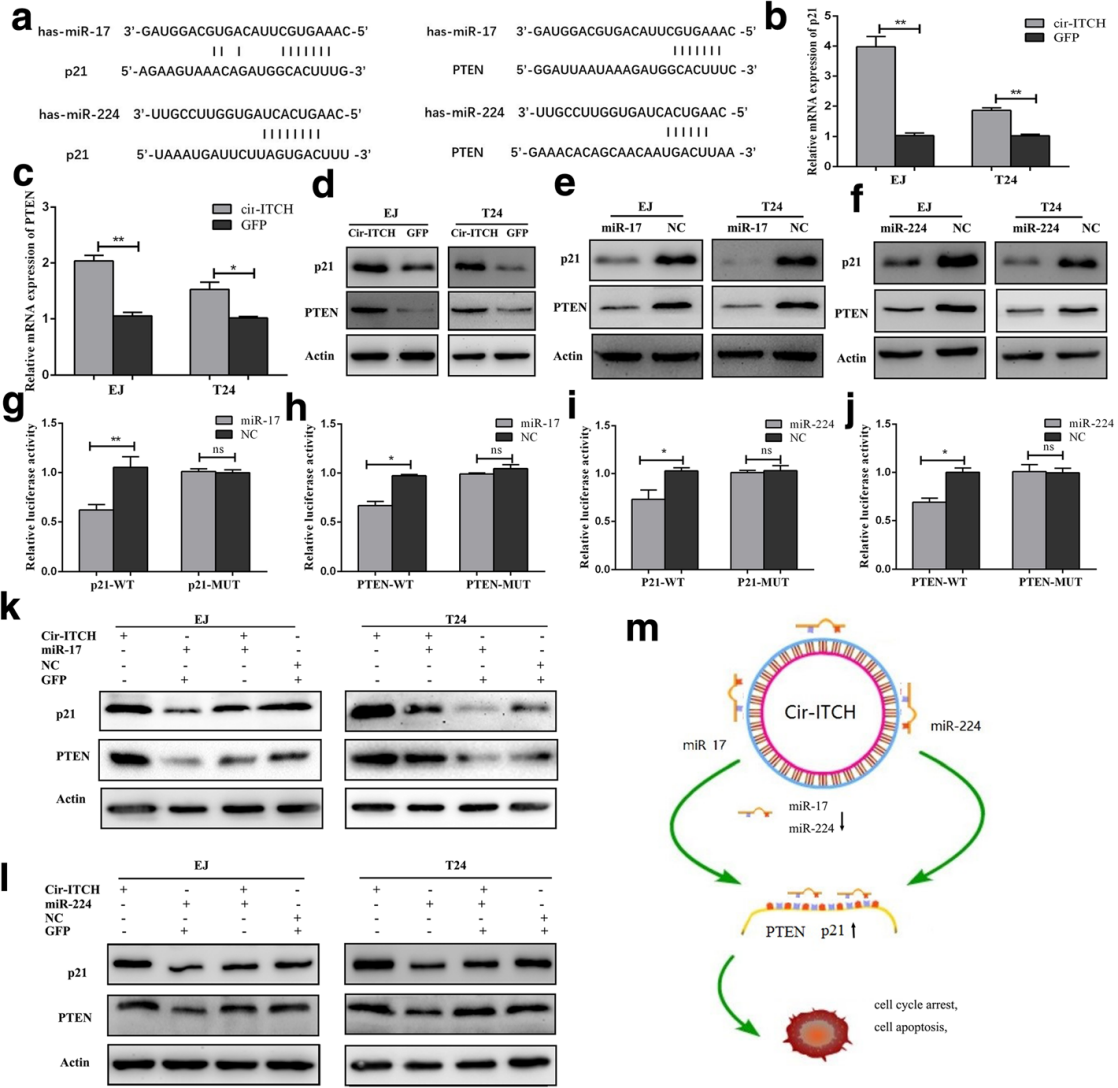
Cir-ITCH modulated the expression of endogenous miR-17 and miR-224 targets p21 and PTEN. **a** Putative miR-17/miR-224 binding sequence in the 3′-UTR of p21 and PTEN mRNA. **b** and **c** cir-ITCH up-regulated the mRNA expression level of p21 and PTEN in BCa cells by qRT-PCR (*P < 0.05, **P < 0.01, Student’s *t*-test). **c** and **d** Circ-ITCH up-regulated the protein expression level of p21 and PTEN by western blot. **e** and **f** miR-17 and miR-224 decreased the protein expression level of p21 and PTEN, individually. **g** and **h** Dual luciferase reporter assays demonstrated that p21 and PTEN are direct target of miR-17(*P < 0.05, **P < 0.01, Student’s *t*-test). **i** and **j** Dual luciferase reporter assays demonstrated that p21 and PTEN are direct target of miR-224(*P < 0.05, Student’s *t*-test). **k** and **l** Western blot showed that mir-17 and miR-224 could partly decrease the protein expression level of p21 and PTEN which were promoted by cir-ITCH. **m** Mode pattern of the cir-ITCH-miR-17/miR-224-p21/PTEN regulatory network

Given that p21 and PTEN share miRNA response elements with circ-ITCH, we next investigated whether miR-17 (or miR-224) targets p21 and PTEN and whether circ-ITCH exerts its anti-tumor effect by modulating the expression of p21 and PTEN (Fig. 6a). As shown in Fig. 6b and c, overexpression of circ-ITCH could increase the mRNA level of p21 and PTEN, and thus increase their protein expression levels accordingly (Fig. 6d). On the contrary, transfection of miR-17 or miR-224 mimics significantly reduced the expression of p21 and PTEN (Fig.6e, f). In addition, dual-luciferase reporter assay was performed. The wild-type 3′-UTR sequence and the mutant 3′-UTR sequence of p21 (or PTEN) were cloned to construct reporter plasmids and mutant vectors, respectively. We found that co-transfection of miR-17 (or miR-224) mimics and reporter plasmids strongly reduced the luciferase activity. Inversely, co-transfection of miR-17 (or miR-224) mimics and mutated vectors showed no obvious effect to the luciferase activity (Fig. 6g-j). Consequently, the findings proved that p21 and PTEN are direct targets of miR-17 (or miR-224).

Besides, the combined transfection of circ-ITCH and miR-17 (or miR-224) was conducted to further assess p21 and PTEN expression. We found that circ-ITCH partly rescued the inhibitory effect of miR-17 (or miR-224) on the expression of p21 and PTEN (Fig. 6k, l), which was agreed with the results of cell function. Taken together, these data demonstrated that circ-ITCH suppressed BCa progression by eliminating miR-17 (or miR-224) oncogenic effect and forming miR-17/miR-224-p21 axis (Fig. 6m).

### circ-ITCH suppressed growth of xenografted tumor in vivo

**Fig. 7:**
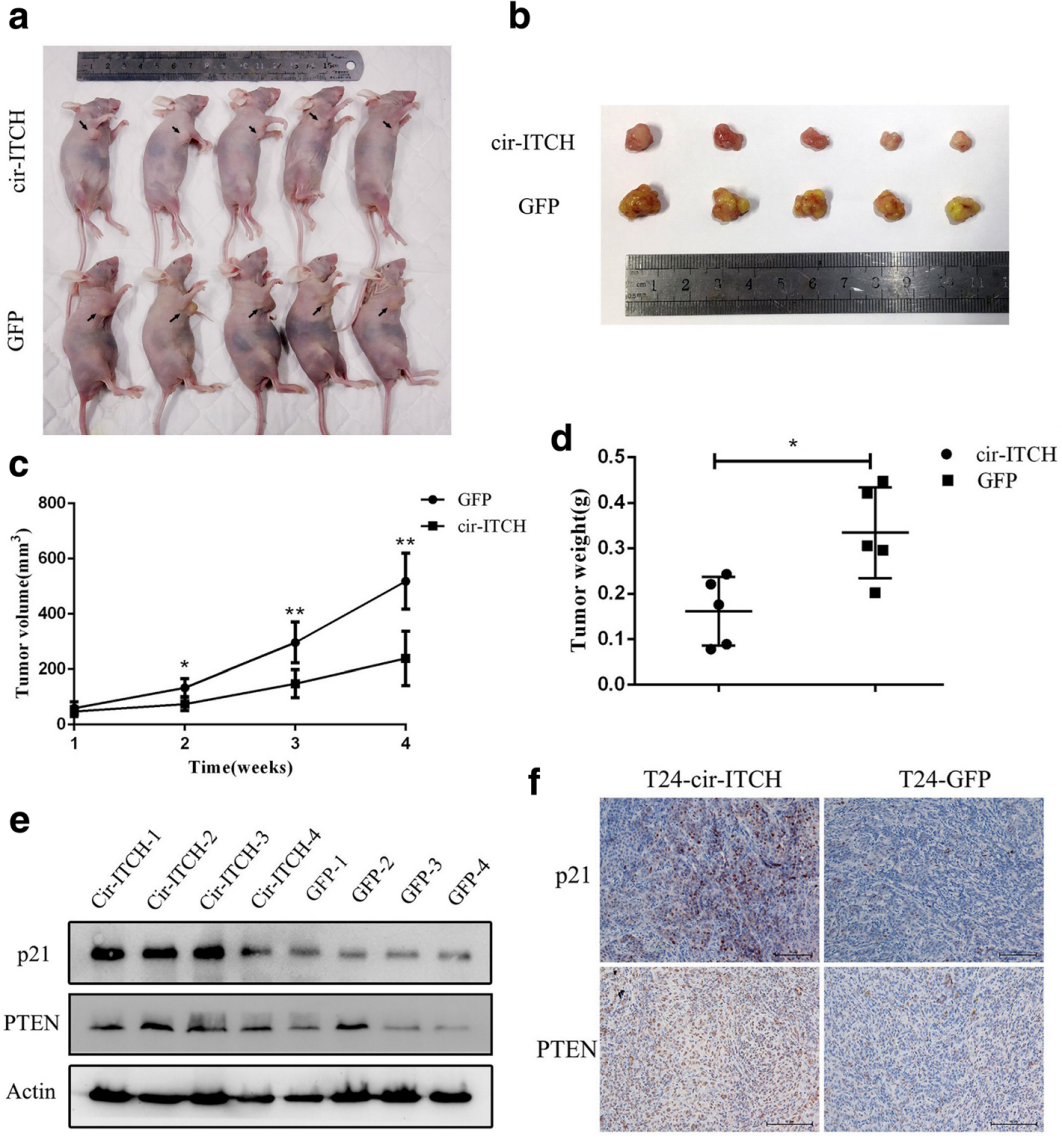
Cir-ITCH suppressed tumor formation of xenograft in nude mice. **a** Representative image of the nude mice injected with T24 cells. **b** Representation picture of tumor formation of xenograft in nude mice (*n* = 5). **c** Summery of tumor volume of mice which were measured every week(*P < 0.05, **P < 0.01, Student’s *t*-test). **d** Weights of tumors in two groups were measured using electronic scales (*P < 0.05, Student’s *t*-test). **e** Proteins was extracted from the tumors and the protein expression of p21/PTEN was measured using western blot. **f** The expression of p21/PTEN was also measured using IHC in xenograft (Magnification, × 200)

To explore whether overexpression of circ-ITCH affects tumor growth in vivo, T24 cells transfected with circ-ITCH or GFP were injected subcutaneously into the nude mice. The tumor volumes were measured weekly after injection. Compared with the GFP group, circ-ITCH overexpression group significantly reduced the mean tumor volume (*P* < 0.05, Fig.7c) and average tumor weight (*P* < 0.01, Fig.7d) than those of GFP group. Western blot and IHC analysis identified that the expression of p21 and PTEN was elevated in the tumors formed from cells overexpressing circ-ITCH (Fig. 7e, f).

## Discussion

In this study, we gradually confirmed the regulatory role of circ-ITCH and its sponging effect for miRNAs in BCa through functional and molecular experiments. The results were totally consistent with our hypothesis that circ-ITCH protects p21 and PTEN and retards BCa progression by sponging oncogenic miRNAs. To our knowledge, this is the first report that generally investigates the expression, function, mechanism and clinical implication of circ-ITCH in BCa.

Specifically, we observed a dramatic decrease of circ-ITCH expression not only in BCa tissues but also in BCa cell lines, suggesting its tumor-suppressive effect. In vitro functional assays showed that circ-ITCH could induce G1 cell cycle arrest and apoptosis, thus inhibiting BCa cell proliferation, invasion and migration. Meanwhile in vivo experiments also supported the tumor-suppressive function of circ-ITCH in BCa. Previous studies revealed that circ-ITCH was down-regulated in ESCC, colorectal cancer and lung cancer and inhibited these tumors progression [11–13]. Taken together, these findings implicated a remarkably tumor-suppressor role of circ-ITCH in most cancers.

Further, we found that overexpression of circ-ITCH increased the expression of p21 and PTEN as expected in our hypothesis, implying a regulatory network between circ-ITCH and p21/PTEN. Notably, cirRNA mainly acts as miRNA sponge and results in a loss of miRNA function accompanied with increased levels of their endogenous targets [25, 26]. For example, CDR1as participates in colorectal cancer and hepatocellular carcinoma by sequestering miR-7 away from its targets [27, 28]. Another cirRNA termed cir-HIPK3, which is derived from exon 2 of the HIPK3, was observed to serve as a sponge for miR-124 [29]. In our study, we screened several predicted oncogenic miRNAs by bioinformatics analysis, and finally confirmed that miR-17 and miR-224 was able to bind with circ-ITCH by biotin-coupled probe pull down assay and biotin-coupled miRNA capture. MiR-17 and miR-224 are crucial molecules targeting anti-oncogenes (including p21 and PTEN) in multiple cancers [30–33]. Further functional studies and luciferase reporter assay also verified that miR-17 and miR-224 promoted tumor progression by directly targeting p21 and PTEN in BCa. Therefore, we presented a novel regulatory axis formed by circ-ITCH-miR-17/miR-224-p21/PTEN in BCa. Additionally, we observed that circ-ITCH did not significantly alter the expression level of its parental gene ITCH in BCa, suggesting that the previous pathway reported in other tumors is not almighty. This discrepancy is complicated and might be caused by tumor heterogeneity.

CircRNAs are promising potential biomarkers because of their unique structure, high stability and specific expression patterns [25]. Though dysregulation of circ-ITCH was frequently observed in human cancers, there is currently few consensus concerning the role of circ-ITCH for the cancer prognosis [34]. In this study, we found that the level of circ-ITCH expression was positively correlated with histological grade and more importantly, circ-ITCH is an independent prognostic marker for survival in patients with BCa. Patients with lower levels of circ-ITCH had a worse overall survival than those with higher circ-ITCH expression. It implied that circ-ITCH might acts as a reliable biomarker and prognosis factor in BCa.

## Conclusions

In summary, our study was the first study to investigate the role and mechanism of circ-ITCH in BCa. The results indicated that circ-ITCH was significantly decreased in BCa and correlated with poor prognosis of BCa patients. Moreover, circ-ITCH suppressed cell proliferation, migration and invasion in vitro and tumorigenesis in vivo. And we suggested a novel circ-ITCH/miR-17, miR-224/p21, PTEN signaling regulatory network in BCa, which may provide a potential biomarker and therapeutic target for the management of BCa.

## Additional files

Additional file 1: Table S1. Primes and probes for PCR and biotin-coupled probe pull down assay and biotin-coupled miRNA capture. (DOCX 13 kb)

Additional file 2: Figure S1. **a** Northern blot with 5 mg of RNA from EJ cells transfected with empty vector (GFP) or circ-ITCH vector (circ-ITCH). The blot was probed against circ-ITCH and 18S ribosomal RNA(loading control). **b**. The overexpression of cir-ITCH had no obvious effect on the expression of its parental gene ITCH using qRT-PCR in BCa cells. **c**. CCK-8 assay showed that knocking down p21 promoted the proliferation ability of BCa cell EJ. **d.** CCK-8 assay showed that knocking down PTEN promoted the proliferation ability of BCa cell EJ. (*P < 0.05, Student’s *t*-test). (ZIP 339 kb)
