# Supplementary material for: Circular RNA circ-ITCH inhibits bladder cancer progression by sponging miR-17/miR-224 and regulating p21, PTEN expression
Source: Mol Cancer. 2018 Jan 31;17:19. doi: 10.1186/s12943-018-0771-7 (PMC5793418; doi:10.1186/s12943-018-0771-7)
Supplement: Supplementary file 1 — Primes and probes for PCR and biotin-coupled probe pull down assay and biotin-coupled miRNA capture. (DOCX 13 kb) [file 12943_2018_771_MOESM1_ESM.docx]

**Supplementary Table s1.** Primes and probes for PCR and biotin-coupled probe pull down assay and biotin-coupled miRNA capture.

| **Primes and probes** | **Sequence** |
| --- | --- |
| cir-itch-forward | 5'-AGCAATGCAGCAGTTT-3' |
| cir-itch-reverse | 5'-TGTAGCCCATCAAGACA-3' |
| ITCH-forward | 5'-AGGATCCCAGGAGTTCAAAT-3' |
| ITCH-reverse | 5'-GAGTGGGCTTGACTGAAATAG-3' |
| β-actin-forward | 5'-AGCGAGCATCCCCCAAAGTT-3' |
| β-actin-reverse | 5'-GGGCACGAAGGCTCATCATT-3' |
| cir-ITCH | 5'-ACAACTACTTCTTCAACCCATCCAGGTGGCAA-3' |
| cir-ITCH-control | 5'-TGTCTGCAATATCCAGGGTTTCCGATGGCACC-3' |
|  |  |
|  |  |
