# Supplementary figures and images for: Circular RNA circ-ITCH inhibits bladder cancer progression by sponging miR-17/miR-224 and regulating p21, PTEN expression
Source: Mol Cancer. 2018 Jan 31;17:19. doi: 10.1186/s12943-018-0771-7 (PMC5793418; doi:10.1186/s12943-018-0771-7)

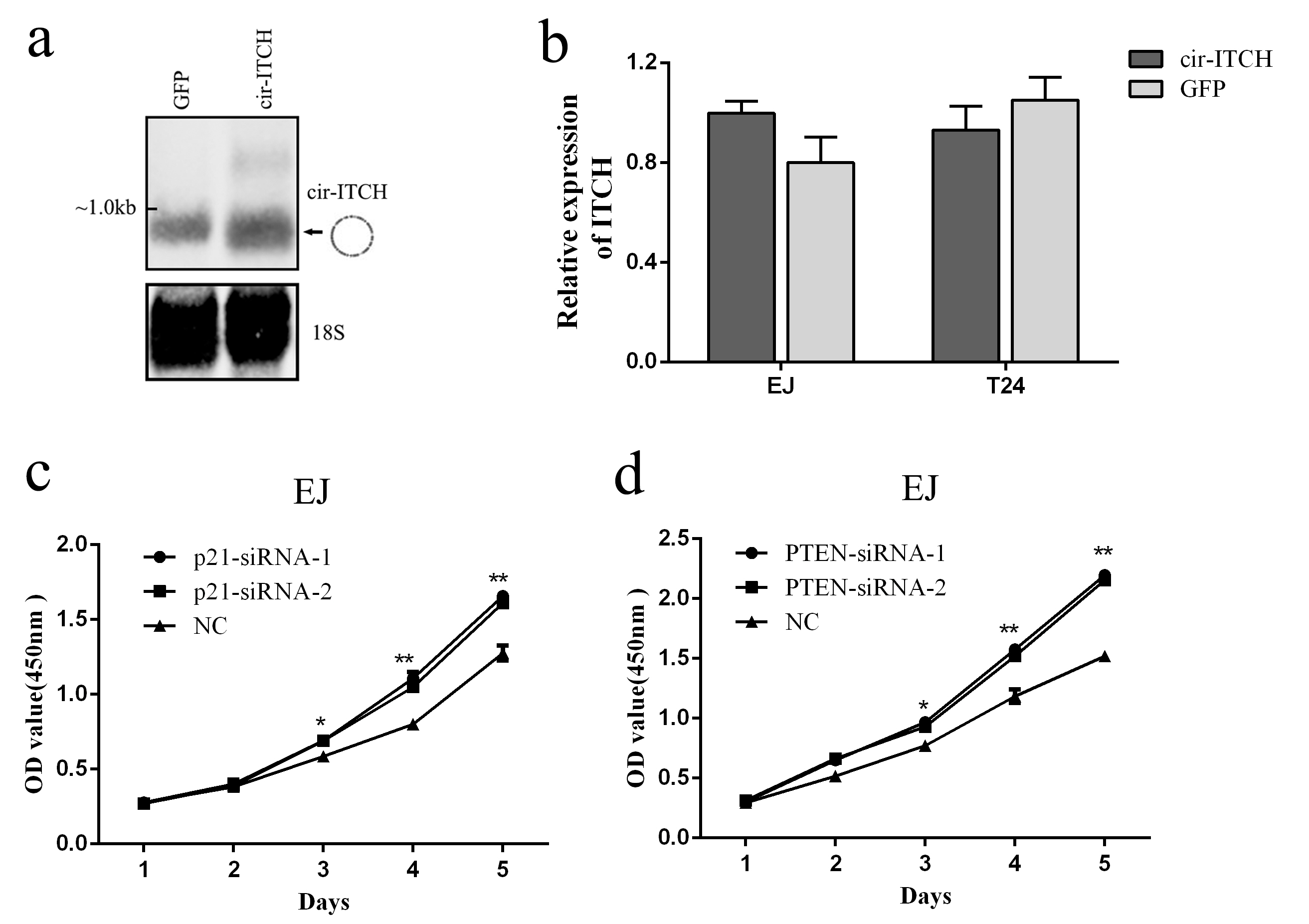

Supplement: Supplementary file 2 — a Northern blot with 5 mg of RNA from EJ cells transfected with empty vector (GFP) or circ-ITCH vector (circ-ITCH). The blot was probed against circ-ITCH and 18S ribosomal RNA(loading control). b. The overexpression of cir-ITCH had no obvious effect on the expression of its parental gene ITCH using qRT-PCR in BCa cells. c. CCK-8 assay showed that knocking down p21 promoted the proliferation ability of BCa cell EJ. d. CCK-8 assay showed that knocking down PTEN promoted the proliferation ability of BCa cell EJ. (*P < 0.05, Student’s t-test). (ZIP 339 kb) [file 12943_2018_771_MOESM2_ESM.zip › Fig S1.png]
